# Brain, Metabolic, and RPE Responses during a Free-Pace Marathon: A Preliminary Study

**DOI:** 10.3390/ijerph21081024

**Published:** 2024-08-03

**Authors:** Florent Palacin, Luc Poinsard, Julien Mattei, Christian Berthomier, Véronique Billat

**Affiliations:** 1 EA 4445—Movement, Balance, Performance, and Health Laboratory, Université de Pau et des Pays de l’Adour, 65000 Tarbes, France; lpoinsard@univ-pau.fr (L.P.); veronique.billat@univ-evry.fr (V.B.); 2 Billatraining SAS, 91840 Soisy-sur-École, France; 3 Physip, 6 Rue Gobert, 75011 Paris, France; j.mattei@physip.fr (J.M.); c.berthomier@physip.fr (C.B.); 4 Faculty of Sport Science, Université Évry Paris-Saclay, 23 Bd François Mitterrand, 91000 Évry-Courcouronnes, France

**Keywords:** marathon, running, electroencephalography, brain activity, cardiorespiratory parameters, exercise performance

## Abstract

The concept of the “central governor” in exercise physiology suggests the brain plays a key role in regulating exercise performance by continuously monitoring physiological and psychological factors. In this case report, we monitored, for the first time, a marathon runner using a metabolic portable system and an EEG wireless device during an entire marathon to understand the influence of brain activity on performance, particularly the phenomenon known as “hitting the wall”. The results showed significant early modification in brain activity between the 10th and 15th kilometers, while the RPE remained low and cardiorespiratory responses were in a steady state. Thereafter, EEG responses decreased after kilometer 15, increased briefly between kilometers 20 and 25, then continued at a slower pace. After kilometer 30, both speed and respiration values dropped, along with the respiratory exchange ratio, indicating a shift from carbohydrate to fat metabolism, reflecting glycogen depletion. The runner concluded the race with a lower speed, higher RPE (above 15/20 on the Borg RPE scale), and reduced brain activity, suggesting mental exhaustion. The findings suggest that training strategies focused on recognizing and responding to brain signals could allow runners to optimize performance and pacing strategies, preventing premature exhaustion and improving overall race outcomes.

## 1. Introduction

Although participation in marathon running has significantly increased [1,2], understanding the physiological responses during a marathon remains a complex challenge. “Hitting the wall“ (HTW) describes the abrupt onset of overwhelming fatigue that often occurs in the later stages of the race, potentially reducing a runner to a walking pace and sometimes preventing them from finishing altogether. Many articles have extensively explored HTW in marathon running [3,4,5,6,7], with the prevailing belief being that runners experience this phenomenon when their glycogen stores become depleted, often due to inadequate race nutrition [8,9,10]. Additionally, poor pacing, whether starting too quickly or finishing too fast, can contribute to suboptimal finishing times [5,9,11,12].

Physiologically, HTW is linked to glycogen depletion, resulting in a less efficient switch to fat metabolism, leading to hypoglycemia and muscle fatigue [13]. Glycogen is a critical energy source during endurance activities, and when these stores become depleted, the body must rely more on fat as an energy source. This switch is less efficient and slower, leading to a reduced rate of ATP production, which is necessary for muscle contractions. Consequently, this can cause hypoglycemia, a condition characterized by low blood sugar levels, and muscle fatigue, as the muscles do not receive adequate energy to maintain the same level of performance [14,15]. This metabolic shift not only impacts muscle function but also affects overall performance by significantly lowering the body’s ability to sustain high-intensity effort. Psychologically, HTW involves central fatigue, where the brain perceives the effort as excessively high. This perception can lead to a decrease in motivation and an alteration in pacing strategies. Central fatigue is associated with the brain’s attempt to prevent harm to the body by reducing the intensity of physical activity when it senses that the physiological demands are too great. This can manifest as feelings of overwhelming tiredness and an inability to maintain pace, regardless of the runner’s physical condition [16,17]. These physiological and psychological factors interplay, creating the characteristic drop in performance. As glycogen stores deplete, the brain receives signals indicating high levels of effort and potential energy shortages. This triggers central fatigue mechanisms, leading to a reduced ability to sustain the previous level of effort, ultimately causing a significant decline in performance [14,16]. The combined effect of muscle energy deficit and central nervous system regulation ensures that the runner slows down or stops, ostensibly to protect the body from further harm [9]. This multifaceted response highlights the complexity of HTW and the intricate balance between physiological energy supply and psychological endurance during marathon running [18,19].

A recent pilot study has provided insights into the cardiorespiratory function during a marathon, using breath-by-breath gas exchange measurements to analyze oxygen uptake ($\dot{V}$O_2_), ventilatory rate ($\dot{V}$E), and their association with the rate of perceived exertion (RPE) throughout the race [20]. This experiment revealed a systematic drift in cardiorespiratory parameters indexed by the RPE ratio. Specifically, RPE was found to increase for a given fraction of maximal cardiorespiratory parameters, indicating that as physiological demand increased, perceived exertion increased disproportionately. The study highlighted that despite RPE being a potential candidate for controlling marathon pace, the indexation of physiological parameters or speed by RPE showed a consistent increase for all runners. This suggests that perceived exertion is not solely dependent on the physiological strain but is also influenced by psychological and experiential factors. Furthermore, this study highlights the multidimensional nature of perceived effort and suggests that even when a runner’s RPE increases more than physiological parameters, it may be associated with a significant change in brain activity during the marathon.

The concept of the “central governor” (CGM) in exercise physiology, introduced by Noakes, provides a theoretical framework for understanding how the brain regulates exercise performance [21]. According to the CGM, the brain continuously monitors and regulates exercise intensity to avoid reaching a state of catastrophic failure or harm, using a combination of physiological, sensory, and psychological feedback for real-time adjustments [16,20]. Electroencephalography (EEG) allows researchers to observe changes in brain activity related to cognitive processes. The utilization of EEG in sports research has traditionally been more prevalent in less dynamic sports [22,23,24]. However, recent advancements in mobile EEG technology and overall technological improvements offer an opportunity to delve into the intricate interplay between neuroscience and sporting behavior, allowing researchers to observe changes in brain activity related to cognitive processes during dynamic sports such as running [25].

The frequency bands commonly explored during exercise and sport research are alpha (α: 8–13 Hz) and beta (β: 13–30 Hz) bands, as they are thought to convey distinct aspects related to motor planning, execution, and control [26,27,28,29,30]. Alpha activity, characterized by quite low-frequency oscillations, is associated with perceptual awareness and the inhibition of non-essential processing, thereby facilitating task performance [31,32]. In contrast, beta activity, characterized by high-frequency oscillations, is associated with voluntary contractions, alertness, and arousal, enhancing the perception of stimuli [33,34,35]. Studies have used the frontal alpha/beta ratio to assess arousal levels, noting that high alpha activity and/or low beta activity are associated with decreased arousal or vigilance [36,37]. In line with these findings, high alpha activity and/or low beta activity in the frontal lobe were observed after running at high intensity [29,38].

To our knowledge, no study has measured real-time brain activity during a marathon. Despite our knowledge of the physiology of running and exercise in general [9,15,39,40], there are still significant gaps in our understanding of how to transpose these results to real-life marathon conditions, where ecological validity is paramount. This research aims to determine whether the brain undergoes more pronounced activity modifications than physiological parameters, and if observable changes in EEG patterns associated with mental fatigue and cognitive decline manifest as the runner nears exhaustion in the last 10 km of a marathon. Monitoring these specific EEG patterns may offer valuable insights into the brain’s cognitive response to the challenges posed by prolonged exercise. We hypothesize that the brain will undergo more pronounced activity modifications than physiological parameters, and that these changes in EEG patterns will reflect mental fatigue and cognitive decline as the runner nears exhaustion. We anticipate observing a decrease in beta activity and an increase in alpha activity [36,37], and/or high alpha activity and/or low beta activity, commonly linked to manifestations of mental fatigue.

## 2. Materials and Methods

### 2.1. Participant

The study involved a single male marathon and trail runner (DB) with nearly 5 years of competitive experience. At the time of the event in 2023, he was 27 years old, 176 cm tall, and weighed 69 kg. The subject volunteered to participate in the study and was asked not to modify his usual training regimen or diet. His marathon record is 3 h, 10 min, and 12 s, set in 2019. He was selected for the homogeneity of his physiological and endurance characteristics [41,42,43]. The subject reported training three to four times per week (50–80 km/week) for over 5 years. His training included high-intensity interval training (6 × 1000 m at 90–100% of his maximal heart rate) once a week and tempo training (15–25 km) at 90–100% of his average marathon speed. DB received information about the study and gave his written consent to participate.

The study’s objectives and procedures were approved by an institutional review board (CPP Sud-Est V, Grenoble, France; reference: 2018-A01496-49). 

### 2.2. Experimental Design: RABIT^®^ Test and the Marathon Race

#### 2.2.1. Pre-Race Protocol

DB performed the RABIT^®^ (Running Advisor Billat Training) test to determine his maximal oxygen consumption ($\dot{V}$O_2_max) and maximal heart rate (HRmax). The RABIT^®^ test was conducted outdoors on a hard dirt path. This test has been validated as a reliable field test of maximal and functional aerobic capacity [44,45]. The RABIT^®^ test, rather than the more commonly used Graded Exercise Test, was chosen because it is based on the RPE pace control.

The test consisted of three incremental exercise stages, adjusted to prescribed RPE levels: “light” (RPE = 11/20) for 10 min, “somewhat hard” (RPE = 14/20) for 5 min, and “very hard” (RPE = 17/20) for 3 min [45]. Exercise intensity was assessed subjectively by the participant, with descriptions ranging from “very light” to “very, very hard”. The RPE scale correlates well with cardiorespiratory and metabolic variables such as minute ventilation, heart rate, and blood lactate levels [46]. Each stage was followed by a 1 min rest period. DB was instructed to adjust his running speed continuously to maintain the prescribed RPE, ensuring that RPE, not speed, remained constant for each stage.

The test revealed DB’s maximal aerobic speed ($\dot{V}$O_2_max) as 18 km.h^−1^, with an HRmax of 179 beats per minute, and a $\dot{V}$O_2_max of 62 mL.min^−1^.kg^−1^.

#### 2.2.2. Marathon Race and Environmental Conditions

Three days after the RABIT^®^ test, DB ran a marathon in Évry-Courcouronnes, France, starting at 10 a.m. The unofficial marathon course consisted of a 5km loop on a flat road. The temperature ranged from 8 to 11 °C (between 10 a.m. and 1 p.m.), with no precipitation and an average humidity of 65%. Blood lactate was measured on the finger (Lactate PRO2 LT-1730; ArKray, Kyoto, Japan) just after the warm-up (15 min at an easy pace) and three minutes after crossing the finish line. Continuous blood lactate measurement was not performed during the race as the ratio of expiratory measurements allowed estimation of the carbohydrate and lipid contribution [47].

### 2.3. Experimental Measurements

#### 2.3.1. Cardiorespiratory Factors and Speed

**Figure 1:**
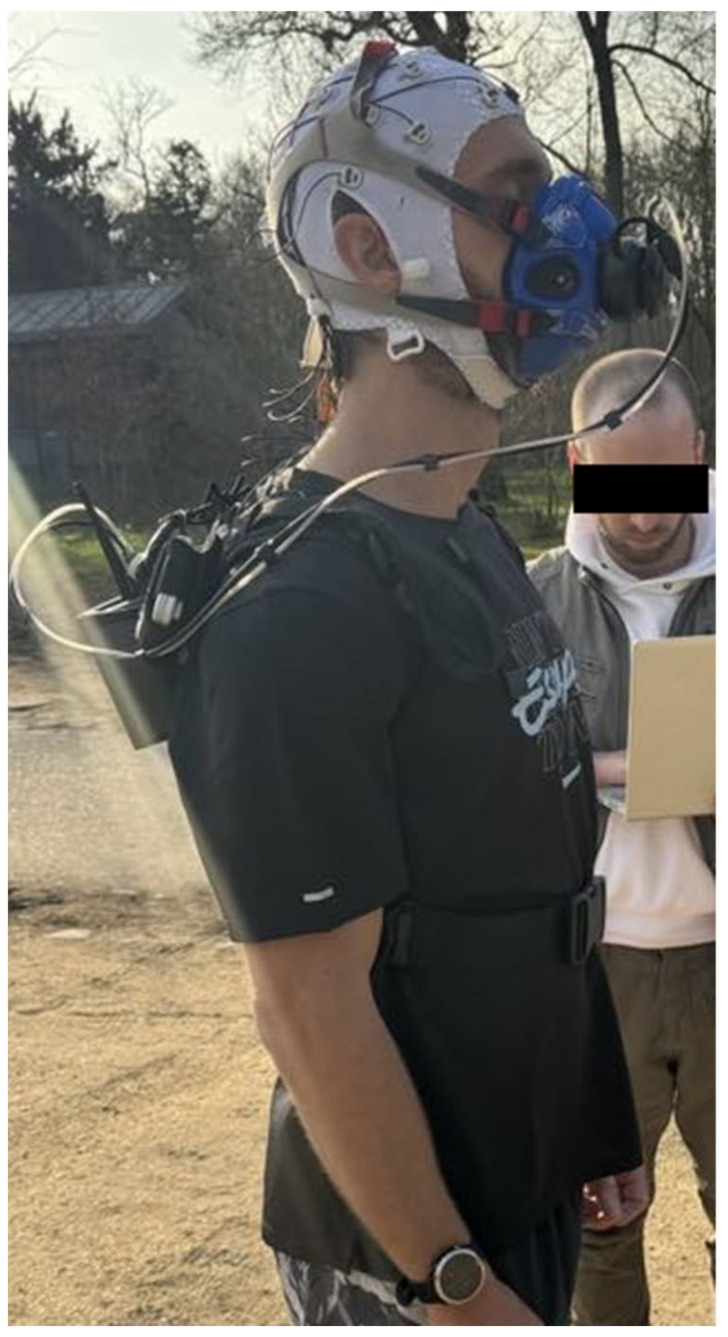
Positioning of different sensors on participant.

Respiratory gasses (oxygen uptake [$\dot{V}$O_2_], ventilation rate [$\dot{V}$E], and the respiratory exchange ratio [RER]) were continuously measured using a telemetric, portable, breath-by-breath sampling system (K5; Cosmed, Rome, Italy) (Figure 1). COSMED reusable face masks are ideal for metabolic testing both at rest and during exercise, regardless of the nature, intensity, or duration of the test. These masks are made of silicone (without latex or other allergenic materials) and are anatomically contoured with a strong ribbed support structure and an integrated chin strap to ensure a perfect fit with no leakage, providing maximum comfort. The runner used the mask version with inspiratory valves to reduce inspiratory resistance during high-intensity exercise for improved comfort.

Additionally, a global positioning system watch (Forerunner 645, Garmin, Olathe, KS, USA) paired with the K5 system was used to measure the heart rate (HR) and the speed response (using 5 s data averages) throughout each trial (Figure 1). The same cardiac belt was used for both the Garmin and K5 systems due to compatibility.

Given that recent research shows marathon performance depends on pacing oscillations [48], runners were encouraged to self-pace their run without focusing on the cardio-GPS, whose dial was hidden. During the marathon, refreshment points (offering water, dry and fresh fruit, and sugar) were located every 5 km as well as at the finish line. At the aid stations, DB was allowed to remove the mask to drink or eat. DB drank one glass at each refreshment point (with flat water and fruits).

#### 2.3.2. The Rate of Perception of Exertion (RPE) Scale

DB was accompanied by an experimenter on a bicycle who showed him the Borg’s 6–20 scale [46] at every kilometer and more frequently if requested by the runner. The RPE was recorded by DB using a small microphone that he carried. DB recorded an RPE at least every kilometer or more frequently if he felt the need. To ensure the accuracy of the report, DB was familiarized with the scale during the 2 weeks preceding the race.

#### 2.3.3. Electroencephalography (EEG) Measurements

EEG was recorded using the LiveAmp 16-channel wireless mobile amplifier connected to gel-based active electrodes (Brain Products, Gilching, Germany) (Figure 1). Due to the straps used to fasten the K5 portable gas exchange system, the posterior electrode sites were not easily accessible. Therefore, referring to the 10–20 extended system, we recorded EEG at the Fp1, Fp2, F7, F3, Fz, F4, F8, FC5, FC1, FC2, FC6, T7, C3, C4, T8, and Pz sites. The REF and GROUND electrodes were placed on the Cz and Fpz sites, respectively. EEG channels were digitized at a rate of 250 Hz, with no filter applied except for the anti-aliasing filter. EEG data were recorded on an exchangeable memory card using the Brain Vision Recorder software (version 1.26.0001, Brain Products, Gilching, Germany).

For EEG processing, artifacts were first detected automatically based on signal power criteria in specific frequency bands. Spectral analysis using FFT over 2 s windows was then performed to obtain spectral power in two conventional frequency bands: alpha α (8–13 Hz) and beta β (13–30 Hz). These values were combined as a sum of ratios to produce an EEG criterion for classifying the runner’s brain activity into several states, ranging from the weakest to the strongest in terms of median and high-frequency power.

### 2.4. Statistical Analysis

Data are reported as means ± standard deviations, unless otherwise stated. 

Given the exploratory nature of this pilot study and the single-participant design, traditional statistical tests to examine differences between kilometer intervals were not applied. The primary objective was to investigate the feasibility of continuous EEG and cardiorespiratory measurements during a marathon and to gather preliminary data that could inform larger, more comprehensive studies in the future. 

Applying statistical tests typically requires a larger sample size to ensure sufficient power and reliability of the results. In the context of a single participant, the variability within the individual’s performance and physiological responses can be high, making it challenging to derive meaningful statistical inferences. As such, the focus was on descriptive analysis and identifying observable trends and patterns in the data.

## 3. Results

### 3.1. Performance (Pacing) and Cardiorespiratory Responses

DB finished the marathon in 3 h, 12 min, and 51 s, very close to his personal best of 3 h, 10 min, and 12 s. 

**Figure 2:**
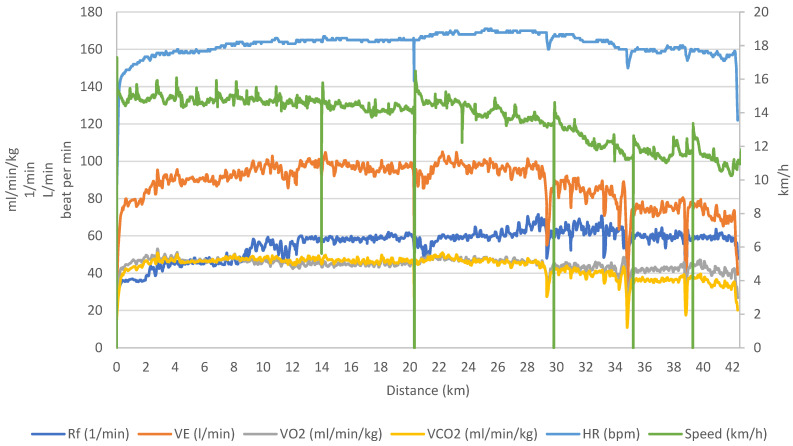
Cardiorespiratory responses and speed during DB’s marathon race. The decline in speed (green curve) at km 14, 20, 30, 35, and 39 corresponds to the refueling stops made by the participant. Rf: respiratory rate, $\dot{V}$E: ventilation rate, $\dot{V}$O_2_: oxygen uptake, $\dot{V}$CO_2_: exhaled carbon dioxide volume, HR: heart rate.

Despite giving 98% effort, DB experienced the dreaded “wall” phenomenon during the race, as shown in Figure 2 (kilometer 30). Running at 76% of his $\dot{V}$O_2_max, 92% of his maximum heart rate, and 72% of his $\dot{V}$O_2_max, DB reached a blood lactate value of 2.5 mM, up from 1.2 mM after the warm-up.

As shown in Figure 2 (and Table 1), his cardiorespiratory responses remained relatively stable from the start until the 30th kilometer. However, beyond kilometer 30, DB hits the “wall”, and there was a noticeable drop in speed and key respiratory parameters, such as $\dot{V}$O_2_, exhaled carbon dioxide volume ($\dot{V}$CO_2_), $\dot{V}$E, and RER. In contrast to the other ventilatory parameters, respiratory rate (Rf) increased until the 12 km mark, then remained stable until the end of the race.

### 3.2. EEG Responses and RPE during the Marathon

**Figure 3:**
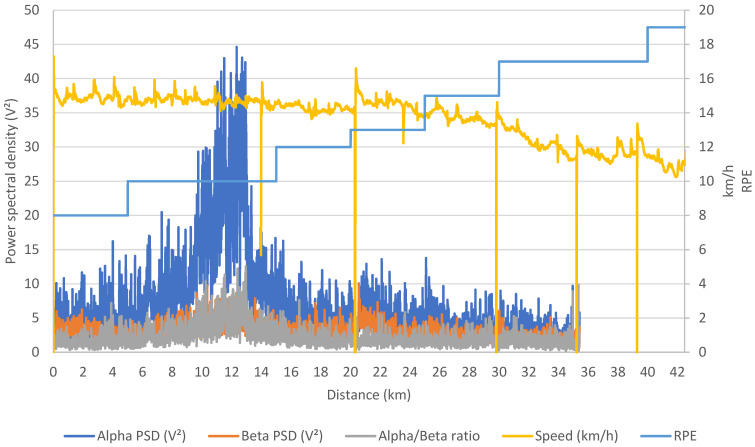
EEG responses and RPE during DB’s marathon race. From the 35th kilometer to the finish line, EEG recording ceased due to battery depletion. The decline in speed (yellow curve) at km 14, 20, 30, 35, and 39 corresponds to the refueling stops made by the participant. PSD: power spectral density, RPE: rating of perceived exertion.

The RPE, illustrated in Figure 3, started at a remarkably low level, registering an initial value of 8/20, indicating a perceived effort categorized as “very easy” according to the Borg scale. However, at the pivotal 26 km mark, coinciding with the start of the HTW, DB’s RPE climbed to 15/20, signifying a transition to the “hard” exercise category. Throughout the final 12 km of the marathon, DB maintained an RPE of 17/20 (with the last km reaching 19/20), corresponding to a “very hard” level of effort.

Figure 3 (and Table 2) shows that significant alterations in EEG response occurred well before DB reached an RPE of 15/20, marking the start of the “hard” phase. Notably, a substantial peak increase in alpha power was observed between the 10th and 15th kilometers, while beta power exhibited only a slight increase. Moreover, from kilometer 26 onwards, a marginal reduction in alpha power was noted, while beta power remained relatively stable.

## 4. Discussion

The present study offers new insights into the concept of the CGM in exercise physiology, as introduced by Noakes [16,21]. This theory suggests that the brain plays a key role in regulating exercise performance by continuously monitoring various physiological and psychological factors. EEG might allow researchers to observe changes in brain activity related to cognitive processes during exhaustive runs. In this case report, we monitored a marathon runner for the first time using a portable metabolic system and an EEG device for the duration of a marathon. Our objective was to investigate whether the brain undergoes more pronounced activity modifications than physiological parameters and to determine if observable changes in EEG patterns associated with mental fatigue and cognitive decline manifest as the runner nears exhaustion in the last 10 km of a marathon. We hypothesized that the brain would show more significant activity changes than physiological parameters and that these EEG changes would be linked to mental fatigue and cognitive decline.

Our results indicated significant early modifications in brain activity between the 10th and 15th kilometers, while the RPE remained low and cardiorespiratory responses were in a steady state (%$\dot{V}$O_2_max and %HRmax). Notably, all EEG frequency bands markedly increased simultaneously at this moment, with the alpha/beta ratio indicating that the alpha band increased more than the beta one. This finding suggests that the runner was in a relaxed but alert mental state. Beta waves, associated with alertness and cognitive processing observed during tasks requiring mental effort, focus, and active thinking [49,50], increased to a lesser extent. These changes occurred while cardiorespiratory parameters and RPE remained stable. This aligns with the CGM, which posits that the brain regulates exercise intensity to prevent harm [16]. Early EEG changes suggest the brain’s anticipatory role in managing effort and maintaining homeostasis.

Notably, significant alterations in EEG responses were observed well before the onset of high RPE and HTW phenomenon, which occurred around the 30th kilometer. The alpha power decreased while beta power remained stable, indicating heightened cognitive effort and mental fatigue. Subsequently, EEG responses decreased after the first hour, surged briefly around the 26th kilometer, and then continued at a slower rate. The alpha/beta ratio was much lower than between the 11th and 14th kilometers. The runner concluded the last hour of the race at a lower speed, higher RPE (above 15/20), and lower brain activity, seemingly in automatic mode. These findings align with the existing literature, highlighting the brain’s role in fatigue and performance decline [9,18].

Additionally, the RER dropped significantly after the 30th kilometer. Initially, the RER was around 1.0, indicating predominant carbohydrate metabolism. However, it dropped to 0.92 between the 30th and 35th kilometers, 0.86 between the 35th and 40th kilometers, and 0.83 in the last two kilometers. This decline in RER signifies a shift from carbohydrate to fat metabolism, suggesting that the runner’s glycogen stores were becoming depleted, leading to increased reliance on fat as an energy source [9,51,52]. This metabolic shift is a hallmark of the “hitting the wall” phenomenon, where glycogen depletion forces the body to rely more on less efficient fat oxidation, contributing to the marked decrease in performance and speed [16,53].

Before directly measuring brain activity during a marathon, which requires specialized expertise in EEG measurement, we conducted a preliminary study to understand the relationship between information obtained from metabolic and biomechanical signals during the marathon. Another preliminary study aimed to explore the hypothesis that entropy, as a measure of uncertainty or randomness, could be associated with the central governor model in several ways [54]. Firstly, the central governor considers the dynamic nature of physiological parameters, such as oxygen consumption, heart rate, and muscle fatigue. Hence, the fluctuations in these physiological factors could contribute to the overall entropy of the system. Secondly, sensory feedback, including proprioception and feedback from muscles and joints, introduces uncertainty into the system. The central governor must interpret and respond to these sensory inputs, and the entropy in sensory feedback contributes to the brain’s challenge in maintaining precise control over exercise intensity. Lastly, psychological factors, such as motivation, perceived effort, and mental fatigue, have been shown to play a significant role in the central governor’s decision-making process. The dynamic nature of psychological states introduces entropy to cognitive control processes, influencing the brain’s regulation of exercise intensity.

With this groundwork, we initiated a case report study, having a runner wear an EEG device during a marathon race while also measuring physiological parameters and speed using a cardiometabolic and portable GPS system. All measures were compared with the runner’s RPE. Our preliminary results suggest that brain activity may be significantly altered during the early stages of the marathon, well before the occurrence of HTW. The pacing strategy did not prevent a decline in speed, suggesting a potential association between EEG activity and mental exhaustion. The changes observed in EEG patterns and the associated increase in RPE support the CGM, indicating that the brain acts as a central regulator of performance by integrating physiological and psychological feedback to prevent damage and optimize effort. While EEG technology offers a direct method to observe these changes, practical applications can be developed for runners to recognize brain signals without such technology. Our findings seem to underline the potential of training strategies that focus on sensation recognition and management, enabling athletes to optimize their performance by adapting to their pace based on cognitive feedback rather than willpower or physiological cues alone. This approach could help runners avoid premature exhaustion and improve their overall performance. However, these preliminary results should be interpreted with caution due to the study’s exploratory nature and the single-participant design. Further research involving larger and more diverse samples is necessary to confirm these findings and expand our understanding of the complex relationship between brain activity, physiological responses, and performance in endurance sports.

## 5. Conclusions and Study’s Limitations

The study tackled the challenge of hitting the wall in marathon running from a novel perspective, exploring this phenomenon through the concept of a central governor intertwined with brain activity. Our preliminary findings suggest a potential link between the CGM and EEG, utilizing the latter to observe and analyze the brain’s electrical activity during exhaustive runs. EEG offers insights into cognitive processes, attention, and fatigue, potentially enhancing our understanding of how the brain’s regulatory mechanisms manifest in real-time during demanding exercise. This integration of physiological and neurological measurements could contribute to a more comprehensive understanding of exercise regulation and fatigue.

Looking ahead, EEG could provide real-time feedback to athletes and coaches on the runner’s cognitive state, helping them to make informed decisions on effort levels and pace adjustments to optimize performance while avoiding premature exhaustion. Recognizing individual variability in the brain’s regulation of exercise, EEG studies could identify unique cognitive responses to fatigue and pacing strategies, contributing to personalized pacing recommendations based on individual cognitive responses.

However, these results should be interpreted with caution due to several limitations inherent in this exploratory pilot study. First, the study was based on a single marathon runner, which limits the generalizability of the results. The observed responses may not represent those of the broader marathon-running population. Additionally, the use of a mobile EEG system and a mask for cardiorespiratory exchange measurements might have influenced the runner’s performance and perceived exertion, potentially introducing biases into the data collected.

As an exploratory pilot study, our work could pave the way for future research. More in-depth studies involving larger and more diverse samples are needed to confirm and develop our findings. Such future research could further elucidate the complex relationship between brain activity, physiological responses, and performance in endurance sports, improving our ability to optimize training and racing strategies.

## Figures and Tables

**Table 1:** Respiratory and speed parameter values at 5 km intervals.

km Interval		Speed (km/h)	Rf (1/min)	$\dot{V}$E (L/min)	$\dot{V}$O_2_ (mL/min/kg)	RER
0–5	Mean ± SD	14.9 ± 0.3	40.8 ± 4.3	84.5 ± 6.7	47.1 ± 2.2	0.95 ± 0.03
	Peak	16.1	47.8	95.5	53.0	1.00
5–10	Mean ± SD	14.8 ± 0.2	48.1 ± 3.8	92.3 ± 3.0	47.0 ± 1.0	1.01 ± 0.02
	Peak	15.9	58.3	100.3	50.3	1.06
10–15	Mean ± SD	14.6 ± 0.5	56.3 ± 3.2	96.9 ± 3.8	45.2 ± 1.3	1.05 ± 0.02
	Peak	15.8	60.0	104.7	47.6	1.10
15–20	Mean ± SD	14.3 ± 0.2	58.8 ± 1.2	96.6 ± 2.0	45.1 ± 1.1	1.03 ± 0.01
	Peak	14.8	61.9	100.5	48.7	1.06
20–25	Mean ± SD	14.5 ± 4.5	57.3 ± 4.0	95.1 ± 7.0	46.9 ± 2.5	0.99 ± 0.03
	Peak	16.6	64.7	105.1	49.8	1.04
25–30	Mean ± SD	13.7 ± 2.4	62.1 ± 3.6	93.2 ± 7.8	45.5 ± 2.8	0.98 ± 0.02
	Peak	14.9	71.5	101.4	48.7	1.02
30–35	Mean ± SD	12.4 ± 0.6	62.0 ± 5.9	81.9 ± 10.3	42.2 ± 4.8	0.92 ± 0.03
	Peak	13.8	70.7	92.1	48.6	0.97
35–40	Mean ± SD	11.1 ± 2.5	58.9 ± 3.8	74.1 ± 6.1	42.1 ± 3.4	0.86 ± 0.02
	Peak	13.4	64.4	80.5	47.1	0.90
40–42	Mean ± SD	11.0 ± 0.4	58.8 ± 2.2	69.0 ± 6.4	40.4 ± 3.1	0.83 ± 0.02
	Peak	11.9	63.5	77.3	46.3	0.86

Note: The shaded line corresponds to the moment when the participant hits the wall. SD: standard deviation, Rf: respiratory rate, $\dot{V}$E: ventilation rate, $\dot{V}$O_2_: oxygen uptake, RER: respiratory exchange ratio.

**Table 2:** EEG and RPE parameter values at 5 km intervals.

km Interval		Alpha PSD (V^2^)	Beta PSD (V^2^)	Alpha/Beta Ratio	RPE
0–5	Mean ± SD	4.7 ± 2.2	3.1 ± 0.7	1.6 ± 0.9	8 ± 0.0
	Peak	16.2	6.3	7.2	
5–10	Mean ± SD	8.5 ± 4.3	3.4 ± 0.9	2.5 ± 1.2	10 ± 0.08
	Peak	29.3	8.6	7.9	
10–15	Mean ± SD	18.2 ± 8.9	4.3 ± 1.3	4.3 ± 2.0	10 ± 0.0
	Peak	44.4	9.8	12.6	
15–20	Mean ± SD	5.5 ± 2.3	2.9 ± 0.8	2.0 ± 1.0	12 ± 0.06
	Peak	16.1	7.2	7.6	
20–25	Mean ± SD	5.2 ± 2.1	3.3 ± 1.2	1.6 ± 0.7	13 ± 0.05
	Peak	13.6	10.1	5.9	
25–30	Mean ± SD	3.9 ± 1.7	2.5 ± 0.8	1.7 ± 0.8	15 ± 0.05
	Peak	13.8	5.9	5.2	
30–35	Mean ± SD	3.1 ± 1.4	2.1 ± 0.6	1.5 ± 0.8	17 ± 0.05
	Peak	9.7	6.1	8.9	
35–40	Mean ± SD				17 ± 0.0
	Peak				
40–42	Mean ± SD				19 ± 0.07
	Peak				

Note: The shaded line corresponds to the moment when the participant hits the wall. From the 35th kilometer to the finish line, EEG recording ceased due to battery depletion. PSD: power spectral density, RPE: rating of perceived exertion, SD: standard deviation.

## Data Availability

The data presented in this study are available on request from the corresponding author.
